# Broad permissiveness of mammalian nectin-4 orthologs for morbilliviruses: a notable exception in dolphin nectin-4

**DOI:** 10.1099/jgv.0.002319

**Published:** 2026-08-13

**Authors:** Mizuki Tominaga, Fumio Seki, Hiroki Nagata, Ayumu Hyodo, Yukiko Akahori, Yuki Kitai, Hiroshi Katoh, Makoto Takeda

**Affiliations:** 1 Department of Microbiology, Graduate School of Medicine and Faculty of Medicine, The University of Tokyo, Bunkyo-ku 113-0033, Tokyo, Japan; 2 Department of Respiratory Viruses, National Institute of Infectious Diseases, Japan Institute of Health Security, Musashimurayama 208-0011, Tokyo, Japan

**Keywords:** Morbillivirus, receptor, nectin-4, host range

## Abstract

Morbilliviruses utilize signalling lymphocytic activation molecule (SLAM) and nectin-4 as their major cellular receptors and exhibit distinct host specificities despite sharing common receptor usage. While species-specific differences in SLAM have been extensively investigated, the contribution of nectin-4 to morbillivirus host range remains poorly understood because nectin-4 is highly conserved among mammals. Here, we systematically compared the receptor activities of nectin-4 orthologs from human, dolphin, bat, dog and seal for representative morbilliviruses, including measles virus (MV), cetacean morbillivirus (CeMV), canine distemper virus (CDV), phocine distemper virus (PDV) and Myotis bat morbillivirus (MBaMV). Cell–cell fusion, virus infection and plaque assays demonstrated that most nectin-4 orthologs efficiently supported infection by all tested viruses. In contrast, dolphin nectin-4 exhibited markedly reduced receptor activity for MV, PDV and MBaMV, while remaining fully functional for CeMV and CDV. This phenotype was consistently observed with multiple clinical MV isolates. Mutational analyses identified amino acid residue 63 as a key determinant of this receptor specificity. Substitution of glutamic acid at position 63 in dolphin nectin-4 with glycine restored MV receptor activity to a level comparable to that of human nectin-4. These findings demonstrate that nectin-4 is generally a broadly permissive morbillivirus receptor but that specific amino acid differences can substantially influence receptor usage. Our results indicate that receptor compatibility contributes to morbillivirus host range and cross-species transmission while being insufficient on its own to fully explain viral host specificity.

**Impact Statement**

Morbilliviruses cause severe diseases in humans and animals and exhibit characteristic host specificities despite sharing the same major cellular receptors. While the contribution of species-specific variation in the immune-cell receptor signalling lymphocytic activation molecule to morbillivirus host range has been extensively investigated, the role of the epithelial receptor nectin-4 has remained unclear because nectin-4 is highly conserved among mammals. Here, we systematically compared nectin-4 orthologs from multiple mammalian species and found that most function as broadly permissive receptors for diverse morbilliviruses. Unexpectedly, dolphin nectin-4 showed markedly reduced receptor activity for measles virus, phocine distemper virus and Myotis bat morbillivirus. We further identified a single amino acid residue, position 63, as a key determinant of this unique phenotype. Our findings demonstrate that even highly conserved receptors can harbour functionally important species-specific differences. These results provide new insight into the molecular basis of morbillivirus host range and emphasize that receptor compatibility cannot be reliably predicted from sequence conservation alone. More broadly, this work advances our understanding of the mechanisms that influence viral host specificity, cross-species transmission and the emergence of morbilliviruses in new host species.

## Introduction

Morbilliviruses include several highly pathogenic viruses that cause serious infectious diseases in humans and animals, such as measles virus (MV), canine distemper virus (CDV), cetacean morbillivirus (CeMV) and peste des petits ruminants virus. These viruses belong to the family *Paramyxoviridae* and possess a non-segmented, single-stranded, negative-sense RNA genome of ∼15–16 kb. They share several characteristic features, including extremely high transmissibility, transient but profound immunosuppression and neuroinvasiveness [1]. Morbilliviruses also share common receptor usage properties. They utilize signalling lymphocytic activation molecule (SLAM/CD150), expressed on immune cells and nectin-4, expressed on epithelial cells, as their major receptors. Thus, members of the genus *Morbillivirus* induce similar pathological manifestations through highly related molecular mechanisms [2, 3].

Nectin-4 is a member of the nectin family of immunoglobulin-like cell adhesion molecules and is predominantly expressed at adherens junctions of epithelial cells, where it contributes to cell–cell adhesion and maintenance of epithelial integrity. Nectin-4 is a type I transmembrane protein consisting of an extracellular region containing one membrane-distal V domain followed by two C2 domains, a single transmembrane segment and a cytoplasmic tail. Among these domains, the membrane-distal V domain directly interacts with the morbillivirus haemagglutinin (H) protein and is therefore the principal determinant of receptor recognition and virus entry.

Despite these common biological features, each morbillivirus generally exhibits characteristic host specificity and causes outbreaks predominantly in particular animal species. For example, MV is maintained exclusively in humans in nature, whereas CeMV circulates among cetaceans and CDV primarily infects carnivores [1, 2, 4, 5]. However, host specificity is not absolute. CDV has been reported in a wide range of carnivorous species and has caused outbreaks in non-human primates [2, 3, 6, 7]. Likewise, CeMV infection has occasionally been documented in pinnipeds [2, 4, 5, 7]. These observations indicate that morbilliviruses can cross species barriers and suggest that contemporary morbilliviruses have evolved through repeated host-switching events during their evolutionary history [8, 9].

Species-specific differences in receptor usage have long been considered important determinants of morbillivirus host range. In particular, SLAM exhibits substantial sequence variation among animal species. Previous studies, including our own, demonstrated that species-specific differences in SLAM contribute to host specificity in certain morbilliviruses [2]. For example, among the viruses examined, only MV efficiently utilized human SLAM [2]. However, many morbilliviruses also efficiently utilize SLAM molecules from multiple animal species and reconstructed ancestral SLAM proteins function as receptors for all morbilliviruses examined [2]. These findings indicate that SLAM usage alone cannot fully explain the characteristic host specificity observed among morbilliviruses.

In contrast to SLAM, nectin-4 is highly conserved among mammals. Amino acid identities exceeding 90% are observed even between evolutionarily distant species, such as humans and dolphins [2]. Consequently, nectin-4 has generally been regarded as a broadly permissive receptor that plays only a limited role in determining morbillivirus host range. However, this assumption has not been systematically tested. Whether species-specific differences in nectin-4 influence receptor usage and contribute to host specificity therefore remains largely unknown.

To address this question, we compared the receptor activities of nectin-4 orthologs from multiple mammalian species for representative morbilliviruses. Our results demonstrate that nectin-4 generally functions as a broadly permissive morbillivirus receptor but also reveal that specific amino acid differences can markedly influence receptor usage in particular virus–host combinations. These findings provide significant insights into the contribution of nectin-4 to morbillivirus host range and cross-species transmission.

## Methods

### Plasmid constructions

The coding sequences of nectin-4 from human, dolphin, bat, dog and grey seal were designed on the basis of the following GenBank entries: human (*Homo sapiens*; NM_030916.3), bottlenose dolphin (*Tursiops truncatus*; XM_033856177.2), greater mouse-eared bat (*Myotis myotis*; XM_036343999.1), dog (*Canis lupus familiaris*; NM_001313853.1) and grey seal (*Halichoerus grypus*; XM_036078881), although the record of grey seal was subsequently suppressed during NCBI genome annotation processing. The dolphin, bat and seal sequences were based on predicted RefSeq transcript models (XM accessions). The corresponding cDNA fragments were commercially synthesized and each synthesized nectin-4 cDNA was cloned into the retroviral expression vector pMXs-IRES-Puro (Cell Biolabs) using standard molecular cloning procedures. The resulting plasmids were designated pMXs-IP-humanN4, pMXs-IP-dolphinN4, pMXs-IP-batN4, pMXs-IP-dogN4 and pMXs-IP-sealN4, respectively.

Since the predicted seal and dolphin nectin-4 sequences possessed unique insertions of several dozen nucleotides, additional pMXs-IP expression vectors carrying modified seal and dolphin nectin-4 cDNAs lacking these insertion sequences were also generated. These plasmids were designated pMXs-IP-sealN4v2 and pMXs-IP-dolphinN4v2, respectively. Site-directed mutagenesis was used to introduce mutations, E63G and N83H, into the pMXs-IP-dolphinN4v2.

Mammalian expression plasmids for the H and F proteins of MV IC-B, CeMV muc, Myotis bat morbillivirus (MBaMV), CDV Ac96I and phocine distemper virus (PDV) 982A strains were previously reported [10].

### Retrovirus production

Retroviral vectors were generated by transfecting the GP2-293 packaging cells with the plasmids pMXs-IP-humanN4, pMXs-IP-dolphinN4, pMXs-IP-batN4, pMXs-IP-dogN4, pMXs-IP-sealN4, pMXs-IP-sealN4v2 and pMXs-IP-dolphinN4v2. Briefly, each plasmid was transfected into GP2-293 packaging cells using a standard transfection reagent according to the manufacturer’s instructions. At 48 h post-transfection, culture supernatants containing recombinant retroviruses were collected. The resulting retroviral vectors were designated Retro-humanN4, Retro-dolphinN4, Retro-batN4, Retro-dogN4, Retro-sealN4, Retro-dolphinN4v2 and Retro-sealN4v2, respectively.

### Cells

P Parental Vero, Vero/hSLAM [11], Vero.DogSLAMtag [12], Vero/dolphin-SLAM (Vero.DolphinSLAMtag) [13], Vero/seal-SLAM (Vero.SealSLAMtag) [13] and Vero.BatSLAMtag [14] were previously reported. HeLa cells were maintained in Dulbecco’s Modified Eagle Medium (DMEM) supplemented with 7.5% FCS and antibiotics (100 U ml^−1^ penicillin and 0.1 mg ml^−1^ streptomycin). They were transduced with Retro-humanN4, Retro-dolphinN4, Retro-batN4, Retro-dogN4 or Retro-sealN4 and stable cell lines expressing each nectin-4 were established by selection with 3 µg ml^−1^ puromycin. They were termed HeLa-Mx-humanN4, HeLa-Mx-dolphinN4, HeLa-Mx-batN4, HeLa-Mx-dogN4 and HeLa-Mx-sealN4, respectively. Vero cells were maintained in DMEM supplemented with 7.5% FCS and antibiotics. They were transduced with Retro-humanN4, Retro-batN4, Retro-dogN4, Retro-dolphinN4v2 or Retro-sealN4v2 and stable Vero cell lines expressing the respective nectin-4 proteins were established by selection with 10 µg ml^−1^ puromycin. They were termed Vero-Mx-humanN4, Vero-Mx-dolphinN4v2, Vero-Mx-batN4, Vero-Mx-dogN4 and Vero-Mx-sealN4v2, respectively.

### Flow cytometry

Cell-surface expression of nectin-4 was analysed by flow cytometry. Cells were harvested and washed with PBS containing 2% FBS, followed by incubation with rabbit anti-nectin-4 polyclonal antibody (HPA010775, Atlas Antibodies) at 4 °C for 30 min. After washing with PBS, the cells were incubated with Alexa Fluor 488-conjugated anti-rabbit IgG secondary antibody at 4 °C for 30 min in the dark. The stained cells were washed twice with PBS and analysed using a BD FACSLyric flow cytometer (BD Biosciences). Data were analysed using FlowJo software. Cell surface nectin-4 expression was evaluated based on the fluorescence intensity of Alexa Fluor 488.

### Viruses

R Recombinant WT MV expressing enhanced green fluorescent protein (IC323-EGFP) has been described previously [15]. Recombinant MBaMV expressing EGFP has been previously reported [16]. CeMV muc strain and PDV 982A strain have been previously reported [13]. The CDV Ac96I strain is also detailed in a previous study [17]. Among the clinical isolates of MV, the H1 genotype virus (MV/Aichi/JPN.9.13) was kindly provided by the Aichi Prefectural Institute of Public Health. The B3 and D8 genotype viruses (MV/Osaka.JPN/17.19 and MV/Osaka.JPN/10.24) were kindly provided by the Osaka Institute of Public Health.

### Virus infection

Sub-confluent monolayers of cells (specific nectin-4-expressing Vero cells and the parental Vero cells) were infected with each morbillivirus at a m.o.i. of 0.01, and the cytopathic effect (CPE) in the cells was observed daily for 4 days.

### Plaque assay

Confluent monolayers of cells (specific nectin-4-expressing Vero cells and the parental Vero cells) were incubated with tenfold serial dilutions of virus samples for 1 h. The cells were then overlaid with DMEM supplemented with 1% methylcellulose and 7.5% FCS (DMEM/MC/FCS). Four days post-infection, DMEM/MC/FCS containing neutral red was added to the cultures. Plaques stained by neutral red were counted the following day.

### Cell fusion assay

Sub-confluent monolayers of HeLa-Mx-humanN4, HeLa-Mx-dolphinN4, HeLa-Mx-batN4, HeLa-Mx-dogN4 and HeLa-Mx-sealN4 cells cultured in 12-well plates were co-transfected with mammalian expression plasmids encoding the H and F proteins of MV IC-B, CeMV muc, MBaMV, CDV Ac96I or PDV 982A strains (0.5 µg each per well), together with the pCA-Neo-AcGFP plasmid (0.5 µg per well). Syncytium formation was monitored daily for 2 days by fluorescence microscopy. For mutational analysis of dolphin nectin-4, sub-confluent monolayers of BHK cells cultured in 12-well plates were co-transfected with pCAG-IC-H and pCAG-IC-F plasmids encoding the MV H and F proteins (0.5 µg each per well), together with the pCA-Neo-AcGFP plasmid (0.5 µg per well) and pMXs-IP-dolphinN4v2 plasmids carrying the E63G and/or N83H substitutions. Syncytium formation was monitored daily for 2 days by fluorescence microscopy.

## Results

### Differential membrane fusion activities of morbilliviruses mediated by mammalian nectin-4 orthologs

**Fig. 1.**
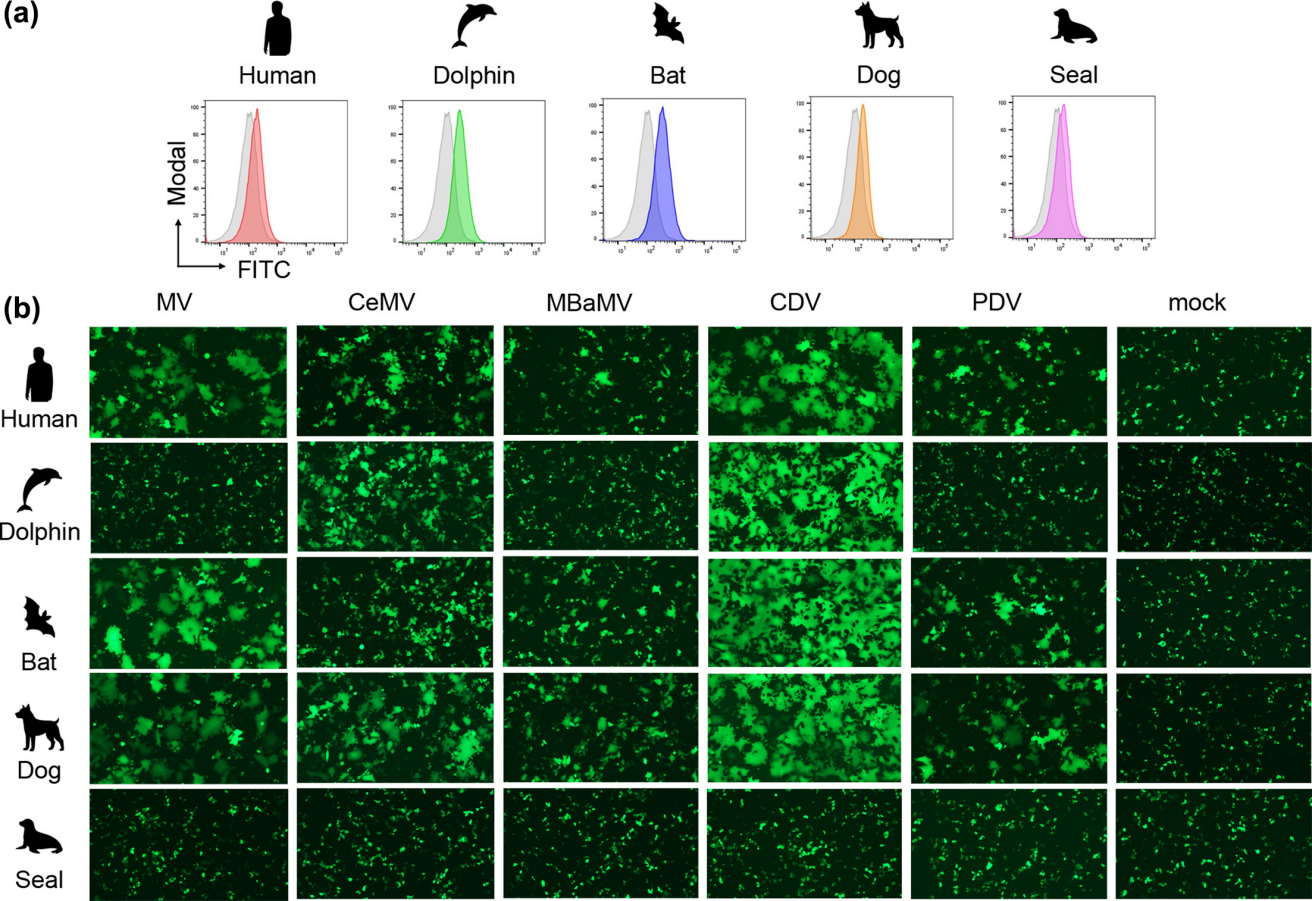
Differential membrane fusion activities of morbilliviruses mediated by nectin-4 orthologs. (**a**) Cell-surface expression of nectin-4 in HeLa-Mx-humanN4 (red), HeLa-Mx-dolphinN4 (green), HeLa-Mx-batN4 (blue), HeLa-Mx-dogN4 (orange) and HeLa-Mx-sealN4 (pink) cells, determined by flow cytometry. (**b**) Representative fluorescence images of syncytium formation induced by morbillivirus H and F proteins. Sub-confluent monolayers of HeLa-Mx-humanN4, HeLa-Mx-dolphinN4, HeLa-Mx-batN4, HeLa-Mx-dogN4 and HeLa-Mx-sealN4 cells were co-transfected with mammalian expression plasmids encoding the H and F proteins of MV, CeMV, MBaMV, CDV or PDV together with an AcGFP expression plasmid. Syncytium formation was monitored by fluorescence microscopy, and representative images obtained at 22 h post-transfection are shown.

Flow cytometric analysis confirmed cell-surface expression of nectin-4 in HeLa-Mx-humanN4, HeLa-Mx-dolphinN4, HeLa-Mx-batN4, HeLa-Mx-dogN4 and HeLa-Mx-sealN4 cells (Fig. 1a). Monolayers of these cells in 12-well plates were transfected with H- and F-protein expression vectors (0.5 µg each) or with empty vectors together with 0.5 µg of an AcGFP-expression vector. At various time points after transfection, multinucleated giant cell formation induced by receptor-mediated cell–cell fusion was observed under a phase-contrast microscope. Membrane fusion activity was also evaluated by fluorescence microscopy based on green fluorescent protein (GFP)-positive syncytium formation. Representative fluorescence microscopic images obtained at 22 h post-transfection are shown in Fig. 1b.

No obvious multinucleated giant cell formation was observed in HeLa-Mx-sealN4 cells expressing the H and F proteins of the tested morbilliviruses (Fig. 1b). In contrast, significant multinucleated giant cell formation was observed in HeLa-Mx-humanN4, HeLa-Mx-batN4 and HeLa-Mx-dogN4 cells expressing the H and F proteins of all tested morbilliviruses (Fig. 1b). In HeLa-Mx-dolphinN4 cells, multinucleated giant cell formation was observed following expression of the CeMV and CDV H and F proteins, whereas no apparent cell fusion was detected in cells expressing the H and F proteins of MV, MBaMV or PDV (Fig. 1b).

### Functional characterization of mammalian nectin-4 orthologs following correction of predicted dolphin and seal nectin-4 sequences

**Fig. 2.**
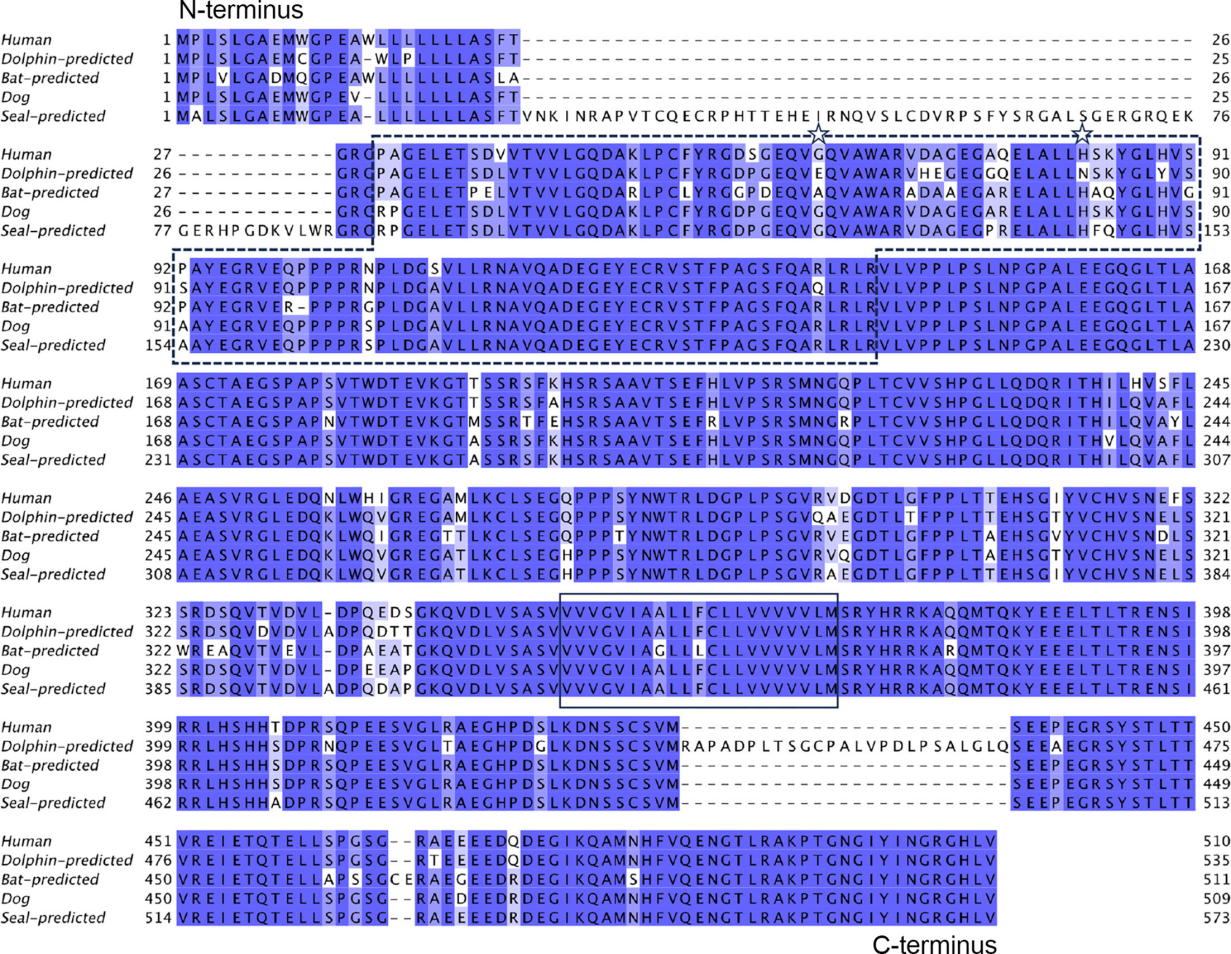
Amino acid sequence alignment of nectin-4 orthologs. Nectin-4 amino acid sequences from human (*Homo sapiens*; NM_030916.3), bottlenose dolphin (*Tursiops truncatus*; XM_033856177.2), greater mouse-eared bat (*Myotis myotis*; XM_036343999.1), dog (*Canis lupus familiaris*; NM_001313853.1) and grey seal (*Halichoerus grypus*; XM_036078881) were aligned. Amino acid conservation is indicated by the intensity of blue shading. The V domain is indicated by a dashed box, and the transmembrane domain is indicated by a solid box. Asterisks indicate amino acid positions 63 and 83.

Comparative amino acid sequence analysis of nectin-4 revealed that two of the predicted sequences, dolphin (XM_033856177.2) and seal (XM_036078881) nectin-4, contained unique insertion sequences that were not observed in the other nectin-4 orthologs analysed. Notably, the insertion sequence was located between the signal peptide sequence and the V domain in the ectodomain of seal nectin-4, while the insertion was located in the cytoplasmic region in the dolphin nectin-4 (Fig. 2).

Because these unique insertion sequences were suspected to modulate the receptor function of dolphin and seal nectin-4, we generated modified constructs lacking the predicted insertion sequences. Although the predicted dolphin insertion was located outside the V domain, it could potentially influence receptor function indirectly by affecting membrane insertion or cell-surface expression. Accordingly, Vero cells expressing modified dolphin and seal nectin-4 lacking the insertion regions (Vero-Mx-dolphinN4v2 and Vero-Mx-sealN4v2) were established.

**Fig. 3.**
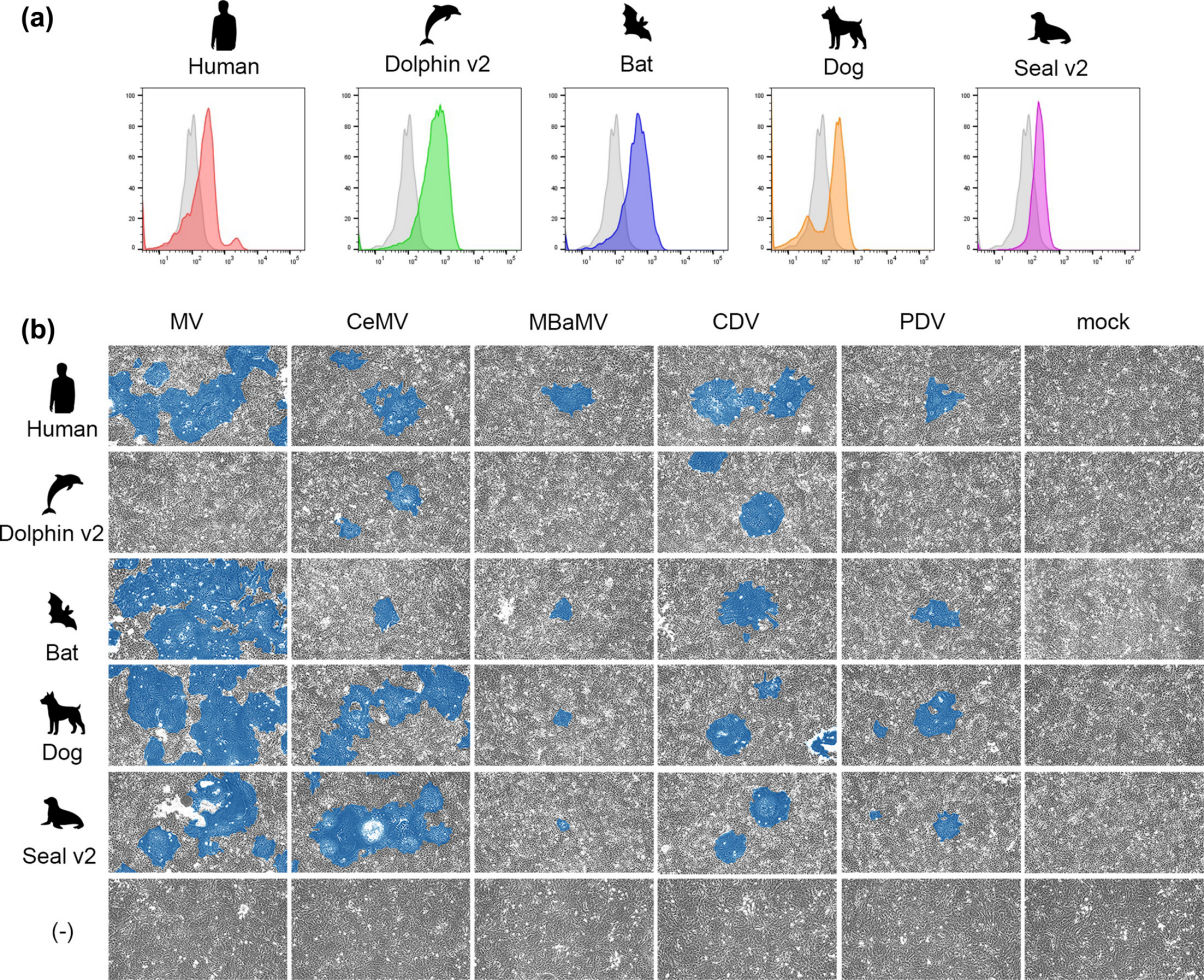
Morbillivirus infection of Vero cells expressing nectin-4 orthologs. (**a**) Cell-surface expression of nectin-4 in Vero-Mx-humanN4 (red), Vero-Mx-dolphinN4v2 (green), Vero-Mx-batN4 (blue), Vero-Mx-dogN4 (orange) and Vero-Mx-sealN4v2 (pink) cells, determined by flow cytometry. (**b**) Representative CPEs following morbillivirus infection. Monolayers of nectin-4-expressing Vero cells and parental Vero cells (−) were infected with each morbillivirus at a m.o.i. of 0.01 and observed daily for 4 days. Representative images obtained at 2 days post-infection are shown. Syncytia are highlighted in blue. The dolphin and seal nectin-4 constructs used in this figure correspond to the modified (**v2**) sequences lacking the insertion regions described in the text.

In parallel, Vero cells expressing human, bat and dog nectin-4 (Vero-Mx-humanN4, Vero-Mx-batN4 and Vero-Mx-dogN4) were also established for comparative analysis. Flow cytometric analysis confirmed cell-surface expression of nectin-4 in Vero-Mx-humanN4, Vero-Mx-dolphinN4v2, Vero-Mx-batN4, Vero-Mx-dogN4 and Vero-Mx-sealN4v2 cells (Fig. 3a).

Monolayers of these cells in 12-well plates were mock-infected or infected with individual morbilliviruses (MV-IC323-EGFP, CeMV muc, MBaMV-EGFP, CDV Ac96I and PDV 982A). In contrast to the cell–cell fusion assay shown in Fig. 1, in which no syncytium formation was observed in cells expressing the originally predicted seal nectin-4, extensive CPEs characterized by syncytium formation were observed following infection with all tested morbilliviruses in Vero-Mx-sealN4v2 cells (Fig. 3b).

When the results were analysed for each virus, similar levels of CPE and syncytium formation were observed in cells expressing human, bat, dog and modified seal nectin-4, but not in the parental Vero cells, following infection with all tested morbilliviruses (Fig. 3b). Thus, efficient syncytium formation required the expression of exogenous nectin-4 orthologs. In contrast, dolphin nectin-4 exhibited a distinct phenotype. Although CeMV and CDV induced clear syncytium formation in Vero-Mx-dolphinN4v2 cells, no syncytium was observed following infection with MV, MBaMV or PDV (Fig. 3b). These results were consistent with those obtained in the cell–cell fusion assay shown in Fig. 1b.

### Quantitative analysis of morbillivirus infection in cells expressing nectin-4 orthologs

To quantitatively evaluate morbillivirus infection mediated by different nectin-4 orthologs, plaque assays were performed using Vero-Mx-humanN4, Vero-Mx-dolphinN4v2, Vero-Mx-batN4, Vero-Mx-dogN4, Vero-Mx-sealN4v2 and the parental Vero cells. Monolayers of each cell line cultured in 12-well plates were infected with 5,000, 500 or 50 p.f.u. of MV, CeMV, MBaMV, CDV or PDV, and plaque formation was assessed after a 5-day incubation period.

**Fig. 4.**
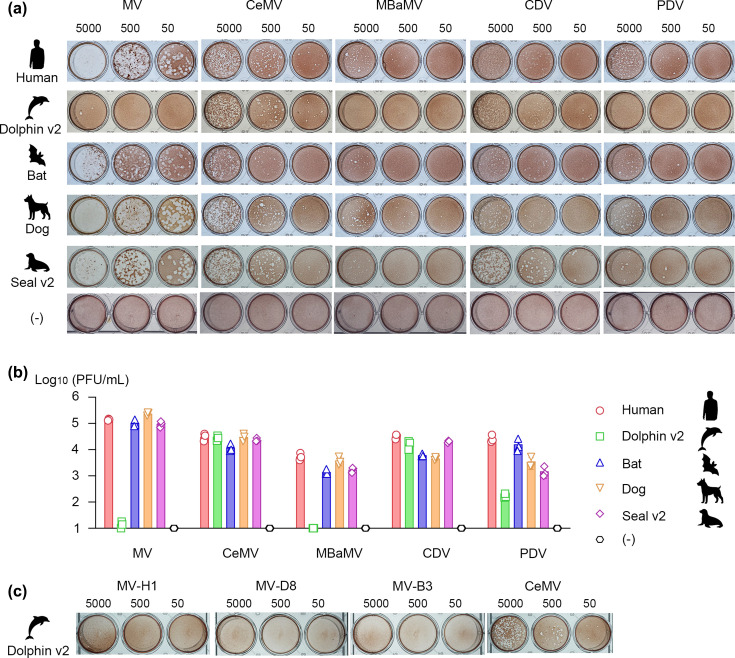
Plaque formation of morbilliviruses in Vero cells expressing nectin-4 orthologs. (**a**) Monolayers of Vero-Mx-humanN4, Vero-Mx-dolphinN4v2, Vero-Mx-batN4, Vero-Mx-dogN4 and Vero-Mx-sealN4v2 cells, as well as parental Vero cells (−), were infected with 5,000, 500 or 50 p.f.u. of MV, CeMV, MBaMV, CDV or PDV and cultured for 4 days in medium containing 1% MC. Plaques were visualized by neutral red staining. The experiment was performed in triplicate, and representative images are shown. Virus inocula were standardized based on infectivity titres determined in Vero cells expressing the corresponding host SLAM receptor for each virus. (**b**) Infectious titres (p.f.u. ml^−1^) determined by plaque assay in Vero-Mx-humanN4, Vero-Mx-dolphinN4v2, Vero-Mx-batN4, Vero-Mx-dogN4, Vero-Mx-sealN4v2 cells and parental Vero cells (−). Stock virus preparations containing 5,000 PFU, as determined in the corresponding SLAM-expressing Vero cells, were subjected to plaque assays on each cell line. Data represent the mean±sd from three independent experiments. (**c**) Monolayers of Vero-Mx-dolphinN4v2 cells were infected with 5,000, 500 or 50 p.f.u. of three clinical MV isolates representing genotypes H1, D8 and B3, together with CeMV and cultured for 4 days in medium containing 1% MC. Plaques were visualized by neutral red staining. The dolphin and seal nectin-4 constructs used in this figure correspond to the modified (**v2**) sequences lacking the insertion regions described in the text.

The results were largely consistent with those obtained in the CPE assays (Fig. 3b). Most morbilliviruses formed plaques efficiently in cells expressing human, bat, dog or seal nectin-4 orthologs but not in the parental Vero cells (Fig. 4a). Thus, efficient plaque formation required the expression of exogenous nectin-4 orthologs. In contrast, Vero-Mx-dolphinN4v2 cells exhibited a distinct phenotype. MV, MBaMV and PDV formed markedly fewer plaques or no detectable plaques, whereas efficient plaque formation was observed in cells expressing the other nectin-4 orthologs (Fig. 4a, b). In contrast, CeMV and CDV formed plaques efficiently in Vero-Mx-dolphinN4v2 cells (Fig. 4a, b).

To determine whether this phenotype was specific to the recombinant MV strain (genotype D3) used in this study, three clinical MV isolates representing different genotypes (H1, B3 and D8) were examined in the same assay. Similar to the recombinant MV strain, all clinical isolates showed markedly reduced or undetectable plaque formation in Vero-Mx-dolphinN4v2 cells, indicating that inefficient utilization of dolphin nectin-4 is conserved among genetically distinct MV strains (Fig. 4c).

### A single amino acid substitution at residue 63 determines the MV receptor activity of dolphin nectin-4

**Fig. 5.**
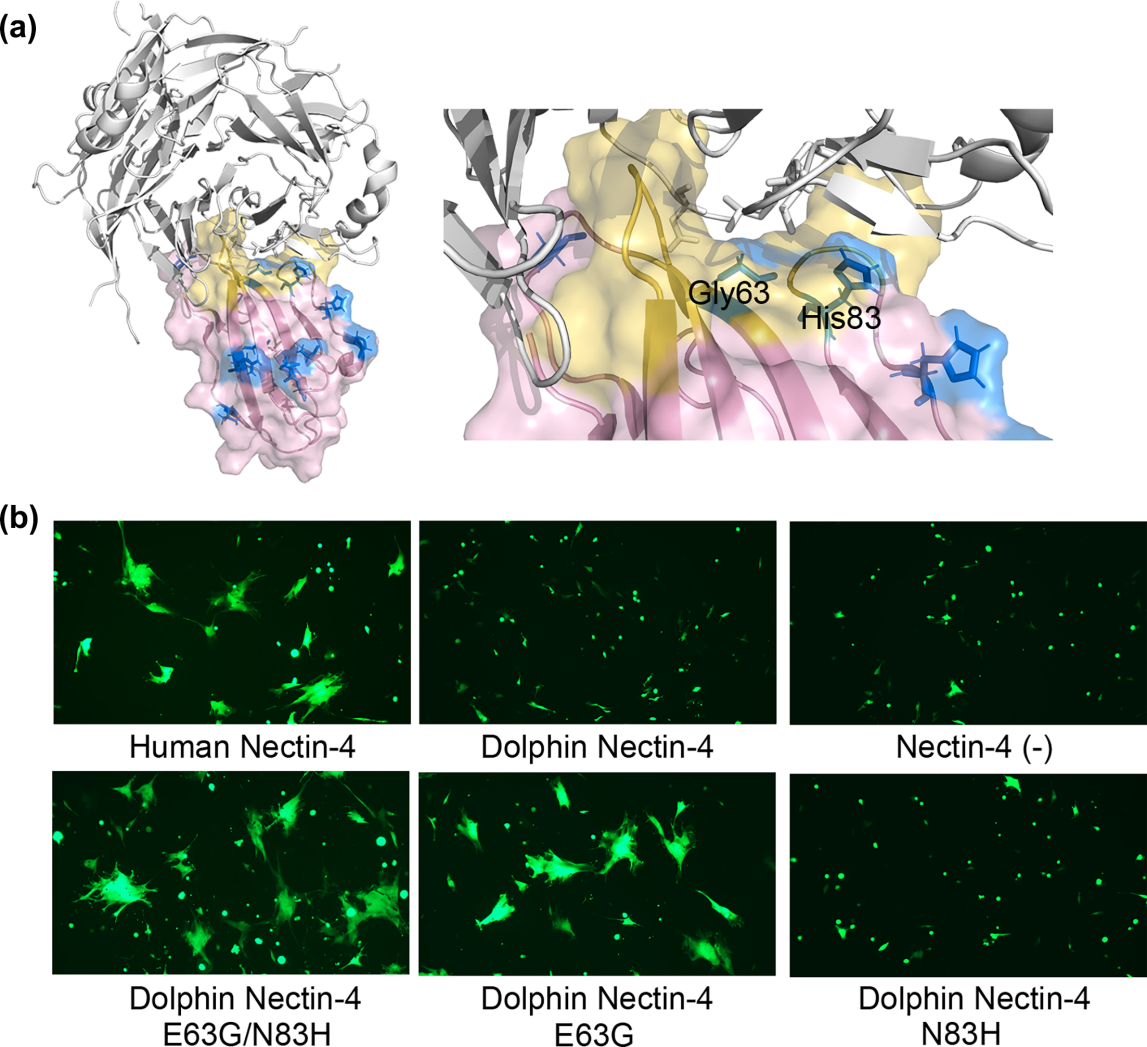
Identification of residue 63 as a determinant of the reduced MV receptor activity of dolphin nectin-4. (**a**) Crystal structure of the complex between human nectin-4 and the MV H protein (PDB ID: 4GJT [18]). The MV H protein is shown in grey and human nectin-4 is shown in pink, yellow and blue. Amino acid residues directly involved in the interaction with the MV H protein are highlighted in yellow, whereas amino acid residues that differ between human and dolphin nectin-4 are highlighted in blue. (**b**) Functional analysis of dolphin nectin-4 mutants. Sub-confluent monolayers of BHK cells were co-transfected with pCAG-IC-H and pCAG-IC-F plasmids encoding the MV H and F proteins, together with an AcGFP expression plasmid and plasmids expressing WT or mutant dolphin nectin-4 carrying the E63G and/or N83H substitutions. Syncytium formation was monitored by fluorescence microscopy, and representative images obtained at 2 days post-transfection are shown.

Comparison of the amino acid sequences of the V domain of human and dolphin nectin-4 revealed 11 amino acid differences between the 2 proteins (Fig. 2). The crystal structure of the MV H protein in complex with the V domain of human nectin-4 has previously been determined [18]. Based on this structural information, we examined the positions of the 11 substituted residues and found that 2 of them were located at sites implicated in the interaction with the MV H protein. These residues corresponded to amino acid positions 63 and 83 (Fig. 5a). At position 63, human nectin-4 contains a glycine residue, whereas dolphin nectin-4 contains a glutamic acid residue. At position 83, human nectin-4 contains a histidine residue, while dolphin nectin-4 contains an asparagine residue. We therefore focused our subsequent analyses on these two residues to determine whether they contribute to the unique receptor characteristics of dolphin nectin-4.

To investigate the contribution of the amino acid differences at positions 63 and 83 to the receptor properties of dolphin nectin-4, a cell–cell fusion assay was performed using BHK cells, which do not express either SLAM or nectin-4. BHK cells were co-transfected with plasmids expressing human nectin-4, WT dolphin nectin-4 or mutant dolphin nectin-4 carrying the E63G substitution, the N83H substitution or both substitutions (E63G/N83H), together with plasmids expressing the MV H and F proteins and a GFP expression plasmid. Then, receptor activity was evaluated by assessing syncytium formation.

As expected, clear syncytium formation was observed in cells expressing human nectin-4, whereas no syncytium formation was detected in cells expressing WT dolphin nectin-4 (Fig. 5b). In contrast, cells expressing the double-mutant dolphin nectin-4 (E63G/N83H) exhibited syncytium formation at levels comparable to those observed in cells expressing human nectin-4 (Fig. 5b). To determine the contribution of each residue individually, the E63G and N83H single mutants were analysed. Syncytium formation was observed in cells expressing the E63G mutant but not in those expressing the N83H mutant (Fig. 5b). Notably, the extent of syncytium formation induced by the E63G mutant was comparable to that observed in cells expressing human nectin-4 (Fig. 5b).

These findings identify amino acid residue 63 as the principal determinant responsible for the reduced receptor activity of dolphin nectin-4 for MV.

## Discussion

Our data indicate that nectin-4 is a broadly permissive epithelial receptor for morbilliviruses, although notable exceptions exist. Because nectin-4-mediated epithelial infection is required for efficient virus shedding and transmission [19, 20], poor use of a host nectin-4 ortholog can represent a host-range barrier [21, 22]. Thus, the main point is not only that most nectin-4 orthologs are functional but also that this broad permissiveness has defined exceptions. Dolphin nectin-4 provides the clearest example of such an exception.

Notably, a single E63G substitution restored the receptor activity of dolphin nectin-4 for MV to a level comparable to that of human nectin-4, identifying residue 63 as a key determinant of receptor function. However, the present data do not distinguish whether this effect results from a direct alteration in H-protein binding or from an indirect change in the local structure or conformational dynamics of nectin-4. Our preliminary structural modelling did not provide a straightforward explanation for the restoration of receptor activity by the E63G substitution (data not shown). Because these analyses were based on static structural models, they may not adequately capture conformational changes associated with receptor engagement and fusion triggering. These observations suggest that the effect of residue 63 may involve conformational dynamics that are not readily captured by static receptor–virus interaction models. Furthermore, because the contribution of residue 63 may differ among morbillivirus H proteins, further structural, biophysical and functional analyses will be required to clarify its role in virus-specific receptor recognition.

These findings of nectin-4, together with our previous SLAM study [14], clarify both the value and the limits of receptor-based explanations of morbillivirus host range. The two receptors differ in their degree of interspecies conservation: SLAM shows substantial interspecies variation, whereas nectin-4 is comparatively conserved [2]. Broad nectin-4 usage is therefore less surprising than broad SLAM usage. In both cases, however, broad receptor usage does not explain natural host range on its own. Receptor usage appears to be most informative when it is inefficient or lost. Inefficient use of human SLAM by non-MV morbilliviruses, inefficient use of bat SLAM by MV and poor use of dolphin nectin-4 by MV, PDV and MBaMV may help explain why particular virus–host combinations are unlikely to support sustained infection or transmission. The dolphin nectin-4 phenotype further suggests that receptor usage cannot be inferred simply from receptor conservation or viral phylogeny, as poor usage was observed across MV, PDV and MBaMV rather than being confined to a single morbillivirus lineage.

The marine morbillivirus system provides a useful context for this interpretation. CeMV has been associated with infections in diverse cetacean species, and sporadic spillover into pinnipeds has also been reported [2, 4, 23–26]. In contrast, PDV is mainly associated with pinniped outbreaks, and PDV infection of cetaceans does not appear to be a prominent pattern among reported marine morbillivirus cases [2, 4, 7, 27, 28]. This epidemiological contrast cannot be attributed to nectin-4 usage alone, because ecological exposure, surveillance intensity, host population structure, immune history and other host factors may all contribute. Nevertheless, our receptor data are consistent with a possible receptor-level contribution: CeMV can use seal nectin-4, whereas PDV shows limited use of the dolphin nectin-4 ortholog tested here. Inefficient use of dolphin nectin-4 may therefore represent one barrier limiting efficient PDV transmission in at least some cetacean hosts.

The converse is also important. When both SLAM and nectin-4 are usable, infection and transmission in nature must depend on additional factors, such as ecological contact, immune restriction, viral replication competence, receptor expression patterns, tissue tropism beyond receptor binding, pre-existing or cross-reactive immunity and transmission dynamics [2, 7, 29, 30]. These additional barriers need to be identified to explain virus–host combinations that appear receptor-compatible but are not observed or are not sustained in nature.

SLAM and nectin-4 play complementary rather than competing roles during morbillivirus infection. SLAM primarily mediates the initial infection and dissemination within immune cells, whereas nectin-4 is essential for epithelial infection, efficient virus shedding and host-to-host transmission. Accordingly, the relative importance of each receptor depends on the stage of infection being considered. Our findings, together with previous studies on SLAM usage, suggest that host range is influenced by the combined compatibility of both receptors rather than by either receptor alone. Thus, asking which receptor is more important may be less informative than understanding how the complementary functions of SLAM and nectin-4 collectively determine viral host range and transmission.

Overall, our findings demonstrate that nectin-4 is generally a broadly permissive morbillivirus receptor, but that specific amino acid differences can substantially influence receptor usage in particular virus–host combinations. These results argue against a receptor-only model of morbillivirus host range while highlighting poor receptor compatibility as a biologically meaningful barrier to sustained infection and transmission. More broadly, our study shows that even highly conserved viral receptors can harbour functionally important species-specific differences, emphasizing the importance of examining receptor function experimentally rather than inferring compatibility solely from sequence conservation.

## Abbreviations

BHK: baby hamster kidney
CDV: canine distemper virus
CeMV: cetacean morbillivirus
CPE: cytopathic effect
DMEM: Dulbecco’s Modified Eagle Medium
H: haemagglutinin
MBaMV: Myotis bat morbillivirus
MC: methylcellulose
MV: measles virus
PDV: phocine distemper virus
SLAM: signalling lymphocytic activation molecule
